# Screening and Genetic Diagnosis of Hemoglobinopathies in Southern and Northern Europe: Two Examples

**DOI:** 10.4084/MJHID.2009.007

**Published:** 2009-08-08

**Authors:** Antonio Amato, Piero C. Giordano

**Affiliations:** 1 Associazione Nazionale Microcitemie Italia (ANMI ONLUS), Centro Studi Microcitemie di Roma, Roma, Italia; 2 Hemoglobinopathies Laboratory, Human and Clinical Genetics Department, Leiden University Medical Center, The Netherlands

## Abstract

Prevention of Hemoglobinopathies has developed around the world based upon the experience done in pioneering endemic countries and is now facing a new phase in non-endemic areas with a recent immigration history. We describe two situations, taking Latium (central Italy) and The Netherlands as two models for endemic and non-endemic countries both confronted with a large multi-ethnic immigrant society. We present prevention results and discuss aspects such as local knowledge and organization. We illustrate the importance of issues like information, carrier diagnostics, screening, counseling and prenatal diagnosis in particular situation of contrasting interest an different ethical opinions. We conclude by underlining the importance of implementing primary prevention at the European level, based upon better information, diagnostics and counseling.

## Introduction:

Hemoglobinopathies (HbP’s) are the most common recessive autosomal disorders in man because carriers are protected against child mortality due to malaria. This advantage has strongly selected the traits in all tropic and subtropic areas of the old world were most carriers live today. However, due to ancient and recent migrations HbP’s are now present virtually everywhere including those non-endemic industrialized areas where people from endemic countries migrate in search of work.

Severe Hemoglobinopathies (Thalassemia and Sickle Cell Disease) are conditions affecting our oxygen transport protein hemoglobin A (HbA). This tetramer consists of 4 globin chains (α2/β2) coded by genes located on chromosome 16 (the alpha genes) and 11 (the beta genes). While β-Thalassemia is caused by defects impairing the expression of the β globin genes, Sickle Cell Disease (SCD) is induced by a single β-gene mutation (HbS), either in homozygous form (HbS/S) or in a number of common compound heterozygous conditions such as HbS/β-thal, HbS/C, HbS/D, HbS/E and others.

The WHO has been recommending primary prevention for many years because these severe diseases, although treatable, cannot be “cured” unless a bone marrow transplant is successfully performed. Therefore primary prevention has been offered for the last 30 years to couples at risk in the Mediterranean area where the incidence of β-Thalassemia major in pioneering countries like Cyprus, Italy and Greece has been reduced to almost zero 1–3.

## Preface

To day also non-endemic countries are in urgent need of prevention because immigrant couples from endemic countries usually do not mix with Northern European, make more children than average and often with a consanguineous partner. All this keeps the chance of getting a child affected by SCD or Thalassemia major as high as in the country of origin^4^.

In the UK, dedicated colleagues were the first who succeeded building up prevention campaigns and to date neonatal screening and early pregnancy diagnostic is available in this country where the expected incidence of the severe conditions is significantly reduced^5^.

In other developed Western European countries with large immigrant populations this problem has been neglected for a long time. Only by the turn of the century public health authorities started to realize that prevention had to be offered to these growing communities but with the exception of Belgium, Northern France and The Netherland, no other continental regions of North European have started offering organized prevention options as yet^6^.

In The Netherlands in particular only a modest incidence reduction has been achieved thus far in spite of substantial efforts in different directions and the implementation of newborn screening (NBS) in 2007^7^. In this country only few couples at risk reach prospective prevention after they have been properly informed and virtually none of those couples identified during NBS because they had an affected child has reached a genetic center thus far^8^.

In contrast, the Thalassemia prevention program applied in the Latium region, the earliest ever planned and introduced in Italy by Silvestroni and Bianco in the early 60^2^, can be considered as the most successful. Since 1993, prospective primary prevention (offered to couples at risk diagnosed and informed before their first pregnancy) was virtually total, reducing the expected incidence of Thalassemia major to virtually zero in the native population. However, due to the many immigrants from endemic countries that are not reached by information and carrier diagnostics, new problems are arising in Latium as well. The situations both in Latium and in The Netherlands were described as two models for endemic and non-endemic countries.

### Methods:

Information and carrier diagnostics are the key elements of prevention. The moment of intervention is crucial and the sooner carriers are detected the better it is for their prevention choices. However, while early, premarital or preconception diagnostics is offered in endemic countries, non-endemic immigration countries have implemented NBS and retrospective prevention for parents of affected children is depending from the advice of pediatricians who may or may not decide to inform the parents about prevention or may or may not refer them to genetic centers.

## The Latium prevention program

The long established prevention campaign consists of a focused biology lesson and universal screening for all pupils attending secondary school. The spin off of this screening consists of information of the parents, carrier diagnostics of parents and family and registration on a data base. Carrier’s follow up concern pre-matrimonial or pre-conception diagnostics, identification of couples at risk and genetic counseling, carrier diagnostics in early pregnancy and prenatal diagnosis after informed decision. Due to the rise of incidence among immigrants the information campaigns has been intensified in order to reach the immigrant population.

## The Dutch prevention attempts

Prenatal diagnosis has been available in the countries from the late 80thies at Leiden reference centre but no prevention campaigns are available. Therefore, all laboratories in the countries have been enquired and instructed how to perform basic carrier diagnostics and how to advice control of parents, partner and sibs, or the younger generation when a infant, a young or an elderly carrier have been diagnosed, respectively. However, to perform carrier diagnostics one needs a doctor prescription, Therefore, many post academic teaching courses and at least one publication a year has been dedicated to enduring education of general practitioners and midwifes over the last 10 years. Explanatory and reassuring letters, addressed to the healthy carriers, explaining the importance of prevention, have been used for all patients diagnosed at the reference centre. First pregnancy control has been offered in a pilot setting and is now implemented in the city of Gouda and is on his way to implementation in the city of The Hague. Neonatal screening has started in 2007. Affected children are referred to dedicated pediatricians in academic centers but referral of the parents to genetic centers remains sporadic. Carrier diagnostics in the preconception phase is slowly growing, mainly based upon medical indication (anemia). Carrier diagnostics based upon ethnic origin is depending from the initiative of informed GP’s eventually responding (or not) to the request of informed patients.

## Technical methods

Carrier diagnostic methods are state of the art protocols in both centers. The simple visual single tube osmotic fragility test^9^ and the erythromorphology evaluation are applied systematically during school screening for Thalassemia in Latium and are part of the carrier diagnostic protocol at Leiden Reference Center, together with the measurement of standard hematological parameters on automatic counters. Both in Latium and The Netherlands separation and estimation of the hemoglobin fractions, either on alkaline electrophoresis or on capillary electrophoresis (Capillarys, Sebia France) and on High Performance Liquid Chromatography (HPLC), is done using different dedicated devices among which the HPLC VARIANT II™ (Bio-Rad Laboratories, Hercules, CA, USA), the HA 8160 (Menarini, Florence, Italy) or the Capillay Electrophoresis device (Sebia, Paris France) as described elsewhere^10^. Ferritin or zink protoporphirine are measured when needed to evaluate iron deficiency.

Molecular analysis is performed in both labs, either routinely or when needed. Genomic DNA is isolated by salt-extraction and point mutation analysis of the β globin gene is done by direct sequencing using commercial devices (ABI Prism, USA). Complex cases are studied by globin chain synthesis 11 and eventually in collaboration 12.

## Results

### Latium:

In the period 2002–2007, 13.597 foreign pupils from 140 different nations, representing the 8.2% of the secondary school population (age 13) were investigated and screened regularly each year. After explaining that school screening is a way to detect healthy carriers and, in due time, couples at risk, 69.4% of the parents responded in favor of prevention sharing the information with their relatives. Screening compliance in immigrants families (64%) was higher than among Italians showing that this initiative is not felt as a stigmatizing issue.

Between 1994 and 2008 a total of 180,477 individuals were examined at the *Ambulatory* Care *Clinic* for carrier diagnostics. The share of foreign subjects visiting our structure has increased in this period from 2.7% in 1994, to 9.74% in 2008.

We have found 8,826 foreign individuals non carriers (of which 2,766 were iron deficient and 6,060 normal) and 2,917 carriers or affected. In the last cohort 22.2% were β-thalassemia carriers, 50.25% were suspected α-thalassemia carriers, 13.06% were HbS, 3.7% HbE, and 1.64% HbC carriers, while 4.18% were carriers of rare Hb variants and 4.97% were affected with severe conditions deriving from associations with HbS, HbC, HbE, α°-thalassemia (HbH disease) and β-thalassemia mutations uncommon in the native Italian population.

**Figure 1.**
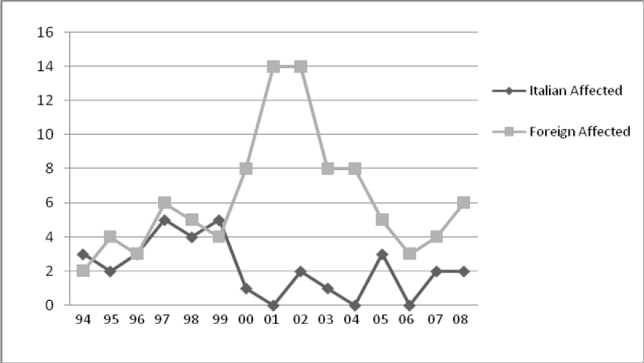
The graph compares the number of affected Italian and foreign patients diagnosed at CSMR for the first time between 1994 and 2008. The rate of foreign increases while the incidence of the Italians currently declines as a consequence of intensive education. Adapted from Amato et al submitted to Prenatal Diagnosis 2009.

In spite of these efforts, the established prevention program does not reach all immigrants and the incidence of non indigenous affected people has increased. Since 2000 practically all affected children are from recent immigrants. The few patients of Italian origin born after 1994 are from well informed couples at risk who refused prenatal diagnosed as a consequence of a personal choice. Data are shown in Figure 1.

### The Netherlands:

Although compared with 10 years ago the improvement is substantial, prevention is still blocked at several bottlenecks in The Netherlands.

Most laboratories in the country are able to provide basic carrier diagnostics 13 but GP’s are still not controlling their foreign clients routinely. Referral for carrier diagnostics is eventually done based upon a diagnostic protocol published by the Dutch GP’s organization advising control when anemia persists after iron therapy^14^. Of course following this protocol most carriers of HbS, C, ad D will remain undiagnosed unless dedicated GP’s will refer upon ethnic indication. Unfortunately, control upon ethnic indication is not yet officially advised by the GP’s organization, while it is for diabetes screening in people of Hindu origin^15^.

It can be estimated that all together no more than 6.000 analyses for carrier diagnostics are annually requested in this way in the country. Considering the natural incidence and the number of demands for prenatal diagnosis, less then 10% prevention has been reached in The Netherlands thus far, meaning that every year about 230 couples at risk do not reach information and miss their chance to choose for prevention.

The National Public Health Council has recently advised more research on preconception screening based on ethnic origin while recently a study at the theoretical level has proposed a strategy based on ethnic origin for combined carrier screening of Cystic Fibrosis (CF) and Hemoglobinopathy^16^, this in spite of the fact that technology and financial effort are totally different.

Early pregnancy control is the most promising initiative. Dedicated midwifes who are taking good care of their clients, advise carrier diagnostics. Pilot studies have been published^17^ and other pilot studies are ongoing hopefully leading soon to a new standard national protocol.

Newborn screening (NBS) has started on January 1^st^ 2007, analyzing near 200.000 samples per year in the same 5 labs which are screening for metabolic diseases. The method of choice (HPLC) was tested at the reference lab in Leiden^18^ and validated in 2006 in 2 screening laboratories^19^–^20^. The method detects at 100% sensitivity SCD and TM and all kind of carriers including alpha thalassemia with elevated Hb Bart’s. Nevertheless, the majority of the TM cases was missed during the first 2 years because some labs went on considering 1–2% HbA normal in non premature newborn (Normal +/− 20%) and did not feed back their results to the reference lab. Meanwhile, incidence figures have been prematurely published with a large underestimation for TM^21^ while an internal discussion is ongoing whether or not to report high HbBart’s and at which level.

In spite of all that, the system is technically sound and the predicted incidence of at least 60 affected children per year was found^22^. Unfortunately, couples at risk, counseled by pediatricians have totally disregarded prevention so far^8^, while enquiries among local parents of affected children have long proved that prevention is welcome in more than 80% of the cases in The Netherlands^23^. Carriers of HbS, C, E, D, O, etc… should be reported for primary prevention of serious Hemoglobinopathies. Unfortunately only HbS carriers have been reported so far, also when parents have chosen to be fully informed. GP’s receive insufficient and unclear information regarding the NBS results and the actions to be taken. Therefore they confuse trait with disease and refer parents of HbS carriers to genetic centers making them unnecessarily worried before any risk is proved. Because of these starting problems, some consider carrier detection “unwanted byproducts” that should not be reported and would prefer to have a method that detects patients only. On the contrary, other think that carriers report should be considered also for other diseases. School screening is not available in the country but should be considered as soon as possible, especially in the large cities.

## Discussion

### Latium:

**Figure 2.**
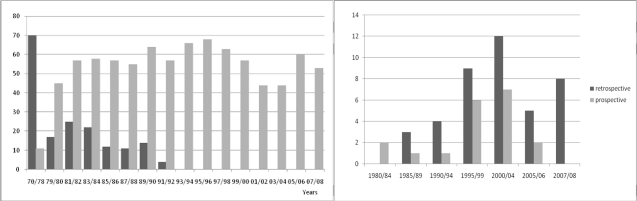
Prospective (grey columns) versus retrospective prevention (black columns) among indigenous couples at risk (left) and immigrant couples (right). Among immigrant couples the prevention it is still largely retrospective, while for Italian couples is fully prospective from 1993. Adapted from Amato et al submitted to Prenatal Diagnosis 2009.

In spite of the good results in offering prospective prevention to the indigenous population (100% from 1994) the prevention for foreign couples at risk have been also in Latium mainly of the retrospective type so far (70%). Out of 60 foreign couples at risk counseled, 42 had retrospectives prevention after at least one affected child was born and only 18 couples had no affected child at the moment of identification. In spite of that our results show that by improving the information campaigns for the immigrant population prospective prevention is possible also among recent immigrants (Figure 2).

### The Netherlands:

The Netherlands has neglected primary prevention for the growing ethnic minorities until the turn of the century 24 and have started to give some attention to the problem only after an enduring process of persuasion 25–30.

Unfortunately, initiatives have not been primarily focused on the implementation of primary prevention strategies and in the end the only official initiative so far (NBS) was taken to provide early treatment as it is the case for Phenylketonuria (PKU). This while these patients do not need treatments shortly after birth, but from 6 months on and therefore do not need to be universally screened at birth but could be better selectively diagnosed during the first semester of life based on the ethnic origin if the GP’s would be available to do that (which is not the case at the moment).

Moreover, the NBS advisory commission is composed mainly by pediatricians, mainly interested in early treatment and much less in carrier analysis and prevention. Contrasting interests are often discussed using ethical concepts, heritage of the conservative politic of the 80thies, based on confusions between recessive traits and late onset dominant genetic diseases. Reasonable in the last case, the right to refuse (genetic) information is improperly applied to the carriers of recessive traits, claiming that healthy carriers could possibly get worried by genetic information and that minors should not be informed until the age of 18, overlooking that in NBS is not the newborn that gets the information but the responsible parents. Under these circumstances primary prevention as a spin off of from NBS has become a tormented history in The Netherlands.

An official implementation of carrier diagnostics in preconception of early pregnancy phase, could also run the risk to become a matter of a long discussion if considered as a national screening. Therefore, carrier analysis should not be considered as a screening to be approved by politics but as a regular diagnostic procedure for which the only approval needed is the one of the informed patient and for which the indication could be a medical one or just the ethnic origin from a high prevalence area. Therefore it is essential to have informed patients requiring carrier diagnostics to informed GP’s and informed labs able to provide the analysis and an explanatory letter.

## Information

It seems evident from our paper that, beside politics, information remains the main problem when implementing prevention strategies. When properly provided, information is providing knowledge and is not producing anxiety or stigmatization to ethnic minorities. Therefore information needs to be offered in a better way and to be adapted to the different cultural situations.

Information in endemic countries is directed to the whole of the population, is better understood, is provided by doctors aware of the situation and nobody is stigmatized. Information in non-endemic countries provided by public campaigns may stigmatize immigrants and should therefore be delivered privately by an educated general practitioner who does not confuses trait with disease and by pediatrician that, beside treating affected children in the best possible way, should refer couples at risk to genetic centers.

A comprehensive and reassuring letter to the diagnosed carrier, to be discussed with the family doctor, is in our experience a way to prevent anxiety and provide correct information to carriers and professional knowledge to the unaware GP. Such a letter is a task for the experienced laboratory providing the diagnosis, both in endemic and non endemic countries. Counseling may need some cultural adaptation. However the fundamental elements of counseling that can be used in all cultures are that i) being carriers is not a disease, ii) that carriers will not develop the disease later on and iii) that knowing which of the recessive traits we carry is an advantage that allows partner control and the prevention of severely affected children.

## Conclusion

Prevention should be encouraged and financially supported at the European level. Prevention should be introduced based on the experiences made both in the pioneering and in immigration countries, both confronted with newcomers, new mutations, new languages and new cultures. Improved information should be provided there where immigrants do not reach prevention through the local information channels. If we do not intervene properly at the European level more severely affected children will be born, having inherited a trait from two healthy parents who have not been informed and who otherwise would have probably chosen for prevention.
